# Efficacy and safety of immune checkpoint inhibitors combined with antiangiogenic agents in advanced cervical cancer: a systematic review and meta-analysis

**DOI:** 10.3389/fimmu.2026.1747768

**Published:** 2026-05-21

**Authors:** Dezhi Zhang, Yuying Meng, Xinyi Dong, Chunming Huang, Xiaolian Ding, Mingmin Wu, Kun Gao, Xin Chang, Yangyi Ou, Min Dong

**Affiliations:** 1 Pharmaceutical College, Guangxi Medical University, Nanning, China; 2 The First People’s Hospital of Fangchenggang, Fangchenggang, China; 3 Guangxi Medical University Cancer Hospital, Nanning, China; 4 Sun Yat-sen University Cancer Center, Guangzhou, China

**Keywords:** anti-angiogenic drugs, cervical cancer, immune checkpoint inhibitors, immunotherapy, programmed death-ligand 1 (PD-L1)

## Abstract

**Background:**

Advanced cervical cancer (aCC) is associated with a poor prognosis for patients. While immune checkpoint inhibitors (ICIs) have demonstrated potential therapeutic benefits, their effectiveness can be hindered by inherent resistance mechanisms and the onset of immune-related adverse events (irAEs). The strategy of combining ICIs with anti-angiogenic agents has gained traction as a potentially effective treatment approach. Nonetheless, there is a notable absence of robust, systematic evidence to confirm the efficacy and safety of this combination specifically for aCC patients.

**Methods:**

We conducted a thorough search of relevant studies across multiple databases, including PubMed, Embase, Cochrane Library, Web of Science, and Scopus. We extracted key outcome measures, such as overall response rate (ORR), disease control rate (DCR), progression-free survival (PFS), overall survival (OS), and adverse events (AEs) for further analysis.

**Results:**

The analysis included three randomized controlled trials (RCTs;all evaluating first-line treatment for persistent, recurrent, or metastatic cervical cancer) and eight single-arm trials(predominantly later-line or mixed-line settings), encompassing a total of 1, 491 patients. The meta-analysis revealed that in RCTs, ICIs significantly improved OS, decreasing the risk of death by 16% (hazard ratio [HR] = 0.84, 95% confidence interval [CI]:0.78-0.91, P <0.001), and enhanced PFS, reducing the risk of disease progression by 18% (HR = 0.82, 95% CI:0.77-0.88, P <0.001). The combination group exhibited a higher objective response rate (ORR, risk ratio [RR] = 1.19, 95% CI:1.10-1.30, P <0.001) compared to the control group. Findings from single-arm studies provided supportive exploratory evidence, showing a pooled ORR of 0.57 (95% CI: 0.46-0.68).Safety profiles were generally acceptable in RCTs, but the absolute rate of severe toxicity in single−arm studies was substantial (51% grade ≥3).Sensitivity analyses affirmed the robustness of the OS results (I² = 0%), with a stable ORR despite observed heterogeneity.

**Conclusion:**

Pooled analysis of first-line RCTs indicates that combining ICIs with anti-angiogenic agents and chemotherapy demonstrates promising efficacy and a manageable safety profile in persistent, recurrent, or metastatic cervical cancer. However, vigilant monitoring and prompt management of immune therapy-related adverse events are crucial for patient safety.

**Systematic Review Registration:**

https://www.crd.york.ac.uk/PROSPERO, identifier CRD420251050867.

## 1 Introduction

Cervical cancer(CC) is ranked as the fourth most common malignant tumor among women worldwide, with approximately 660, 000 new cases diagnosed in 2022 (1). The treatment landscape for CC is evolving rapidly, particularly in the management of aCC, which encompasses both locally advanced cervical cancer (laCC), classified by the FIGO 2018 staging system as IB3, IIA2–IVA, and recurrent or metastatic cervical cancer (r/mCC). For laCC, concurrent chemoradiotherapy (CCRT) remains the standard treatment. In contrast, systemic therapy is the primary approach for r/mCC (2, 3). Despite these treatment strategies, the 5-year survival rate for aCC remains under 20%. For instance, the median overall survival (mOS) reported in the GOG-240 trial, which established a first-line standard treatment regimen of bevacizumab combined with chemotherapy, was only 16.8 months (4). As a result, ongoing research is continuously reshaping the diagnostic and therapeutic frameworks for aCC.

A significant breakthrough in tackling the treatment challenges associated with aCC has been the introduction of immunotherapy (5). In 2023, a wealth of clinical trial results emerged, signaling the beginning of a new era in aCC treatment. The KEYNOTE-158 trial demonstrated that pembrolizumab monotherapy achieved an ORR of 14.3%, which improved to 17.1% among patients with PD-L1 positive tumors (6). Given this relatively modest efficacy, researchers are increasingly exploring combination treatment strategies. Among these, the combination of ICIs with vascular- targeted therapies has shown substantial promise in clinical trials. This approach seeks to normalize tumor vasculature, modulate the immunosuppressive tumor microenvironment (TME), and enhance the infiltration of effector T-cells, thereby synergistically activating the immune system to recognize and eliminate tumor cells (7, 8).

Currently, numerous key clinical trials have assessed the clinical value of combination therapy strategies. One notable example is the BEATcc trial, which investigated the efficacy of the PD-L1 inhibitor atezolizumab in conjunction with the standard treatment regimen established in the GOG240 trial, comprising bevacizumab and chemotherapy (9). This trial successfully met its primary endpoint, revealing significant and clinically relevant improvements in both PFS and OS. Specifically, the trial reported a median PFS (mPFS) of 13.7 months and a mOS of 32.1 months. These outcomes surpassed those of the GOG240 trial, which recorded an mPFS of 8.2 months and an mOS of 17.0 months, as well as the KEYNOTE-826 trial, which reported an mPFS of 10.4 months (10). However, direct cross−trial comparisons should be interpreted with caution because of differences in patient populations, treatment backbones, PD−L1 thresholds, and eras of conduct. Furthermore, the COMPASSION-16 study found that cadonilimab, a PD-1/CTLA-4 bispecific antibody combined with chemotherapy, offered survival benefits even for patients with PD-L1 negative status, as indicated by a hazard ratio [HR] of 0.77 (11). Despite these encouraging results, they have yet to be incorporated into systematic reviews, and ongoing debates persist regarding variations in treatment effects based on differing PD-L1 expression levels, long-term safety profiles, and the most effective drug combinations.

To address these gaps, this meta-analysis systematically evaluates the efficacy and safety of combining immune checkpoint inhibitors with anti-angiogenic agents for treating advanced cervical carcinoma. The analysis will focus on several key questions: (1) Does combination therapy significantly enhance OS and PFS compared to standard treatment? (2) Are patients with PD-L1 negative status able to benefit from this approach? (3) Do various anti-angiogenic agents, such as monoclonal antibodies versus TKIs, exhibit differences in efficacy and safety? This review focuses only on recurrent or metastatic cervical cancer (r/mCC). All included studies enrolled patients with persistent, recurrent, or metastatic disease. LaCC was not covered by the pooled analyses. The insights gained from this analysis are expected to provide robust evidence for clinical practice and inform individualized treatment strategies.

## 2 Materials and methods

This study is a systematic review and meta-analysis conducted in strict accordance with the PRISMA (Preferred Reporting Items for Systematic Reviews and Meta-Analyses) (12) guidelines and the Cochrane Handbook. The protocol for this study has been registered with the PROSPERO international prospective register of systematic reviews (Registration number: CRD420251050867).

### 2.1 Study design and search strategy

We conducted a systematic literature search across several electronic databases, including PubMed, Web of Science, Cochrane Library, Embase, and Scopus, with the final search date set for April 30, 2025 (unified throughout). The retrieval process involved a combination of subject headings and free-text terms, tailored to the specific requirements of each database. Our search strategy was structured into three main components: (1) Terms related to “immune checkpoint inhibitors, “ such as “immune checkpoint inhibitors, “ “PD-1 inhibitors, “ “PD-L1 inhibitors, “ “nivolumab, “ “pembrolizumab, “ “sintilimab, “ “camrelizumab, “ “toripalimab, “ “tislelizumab, “ “atezolizumab, “ “durvalumab, “ and “avelumab”; (2) Terms associated with “anti-angiogenic drugs, “ including “anti-angiogenic drugs, “ “bevacizumab, “ and “anlotinib”; and (3) Terms related to “Uterine Cervical Neoplasms, “ specifically “Uterine Cervical Neoplasms” and “cervical cancer.” Our search spanned from the inception of the databases until April 30, 2025, with a comprehensive search strategy detailed in **Supplementary Data 1**.

### 2.2 Inclusion and exclusion criteria

To qualify for inclusion, studies had to report at least one of the predefined endpoints: ORR, DCR, PFS, OS, or AEs. Tumor responses were evaluated based on the RECIST version 1.1 (13, 14), and, while the incidence and severity of adverse events were assessed using the Common Terminology Criteria for Adverse Events (CTCAE) (15). The exclusion criteria were as follows: (1) meta-analyses, reviews, retrospective studies, case reports, conference presentations, editorials, and studies conducted on *in vitro* or animal models; and (2) studies that were off-topic, contained incomplete or duplicate data, or were non-English studies without an official English translation.

### 2.3 Data extraction and quality assessment

Data extraction and quality assessment were performed collaboratively by two researchers, Dezhi Zhang and Xinyi Dong, utilizing a pre-designed standardized form. Any discrepancies encountered were resolved through discussions with a third researcher (Chunming Huang). The data extracted included various study characteristics (author, year, country, study design, and phase), patient demographics (sample size, age, FIGO stage, histology, PD-L1 expression), treatment regimens (types of immune checkpoint inhibitors, anti-angiogenic drugs, chemotherapy regimens), and efficacy and safety endpoints (OS, PFS, ORR, DCR, incidence of AEs, and grade ≥3 AEs). For RCTs, the Cochrane risk of bias assessment tool was employed, while the ROBINS-I tool was utilized for single-arm studies (16–18). Although ROBINS−I was originally developed for non−randomized comparative studies, we chose it for single−arm studies because there is currently no widely accepted, validated tool specifically designed for non−comparative single−arm prospective trials. We used it to assess potential bias due to confounding, selection, and reporting. This choice is acknowledged as a limitation of our study. High-risk studies were excluded from sensitivity analyses to enhance the robustness of our findings.

For the three included RCTs, we applied the following subgroup extraction strategy to ensure a consistent anti−angiogenic comparison: in the BEATcc trial, all patients received bevacizumab, so full trial data were used. In KEYNOTE−826 and COMPASSION−16, where bevacizumab was not mandatory, we extracted the pre−specified subgroup data from the original reports comparing patients who received bevacizumab plus ICI plus chemotherapy versus those who received bevacizumab plus chemotherapy alone. This allowed us to pool results to compare “chemotherapy + bevacizumab + ICI” versus “chemotherapy + bevacizumab (without ICI)”.

### 2.4 Statistical analysis

In this meta-analysis, we analyzed data from RCTs using Review Manager Version 5.4 (RevMan 5.4), while data from single-arm studies were combined using STATA 18.0 (Stata Corp LLC, College Station, TX, USA) for a single-group rate meta-analysis (19). For single−arm studies, pooled proportions (ORR, DCR, and AE rates) were calculated using a random−effects model with the logit transformation to stabilize variances. We assessed between-study heterogeneity using the I² statistic, applying a random-effects model for I² values of 50% or higher. We explored potential sources of heterogeneity through sensitivity analyses and subgroup stratifications based on treatment regimen, PD-L1 expression status, and histological subtype. Given the substantial clinical heterogeneity in lines of therapy (first−line vs. later−line) and backbone regimens (with vs. without chemotherapy), we prespecified that results would be reported separately for RCTs (first−line) and single−arm studies (later−line/mixed−line) rather than calculating a single pooled estimate across all studies.

## 3 Results

### 3.1 Study selection

**Figure 1:**
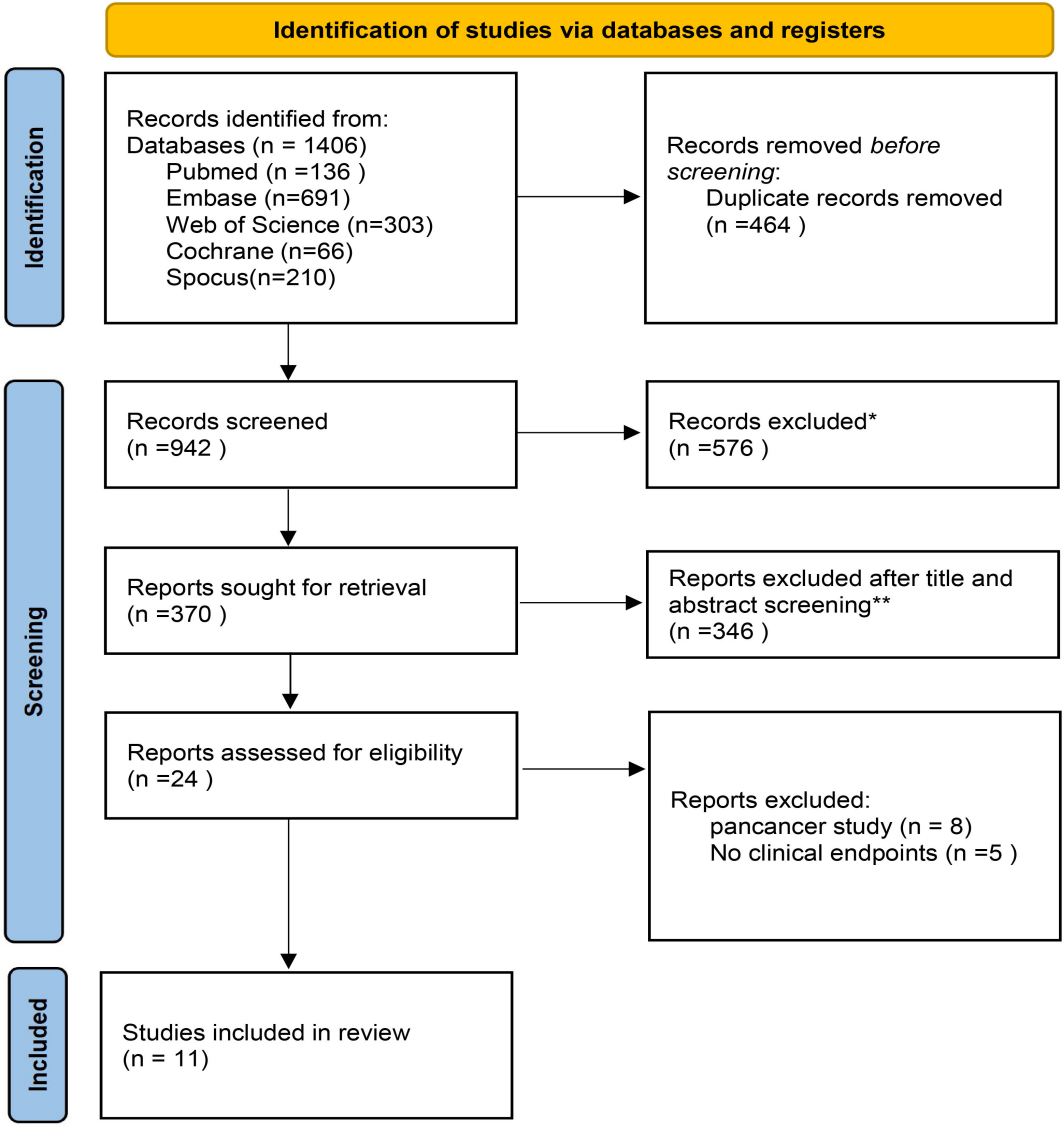
PRISMA flowchart of the screening and inclusion process. *Records excluded: Case reports (n=78); meeting abstract (n=33); Editorial, Note, personal opinions and letter (n=18); Reviews (n=392); meta or cost-effectiveness analysis(n=37);no English(n=18); **Reports excluded after title and abstract screening (n=230): animal or Basic research(n=83); No cervical cancer (n=122); No immunotherapy-Targeted therapy (n=57); Retrospective study(n=50); Updated/more detailed report of same trial available (n=34).

Our search yielded 1, 406 studies across various databases, including 136 from PubMed, 691 from Embase, 303 from Web of Science, 66 from the Cochrane Library, and 210 from Scopus. After removing 464 duplicates, we screened the titles and abstracts of the remaining studies. Full-text reviews were conducted for 24 articles, leading to the inclusion of 11 studies that met our predefined criteria. A flowchart illustrating the search strategy is shown in **Figure 1**.

### 3.2 Study characteristics

This systematic review encompasses 11 studies, comprising 3 RCTs (9, 11, 20) and 8 single−arm (21–28) trials. All three RCTs evaluated first−line treatment for persistent, recurrent, or metastatic cervical cancer (r/mCC) using ICIs combined with bevacizumab plus platinum−based chemotherapy. The eight single−arm trials predominantly enrolled patients with later−line or mixed−line advanced, recurrent, or metastatic cervical cancer, with interventions including ICIs combined with tyrosine kinase inhibitors (anlotinib, famitinib, apatinib, axitinib) or, in a few studies, bevacizumab. Detailed characteristics of the studies and baseline patient data are available in **Table 1** and the **Supplementary Materials** (**Supplementary Table**).

**Table 1:** Characteristics of the included studies.

Study name (trial)	Phase	Line of therapy	Disease setting	Chemotherapy backbone	Anti-angiogenic agent (type)	ICI	N	PD-L1 expression	Histological subtype (n)	Key endpoints
RCT										
Oaknin A 2024 (9) (BEATcc/ENGOT-cx10)	III	First-line	Persistent, recurrent, or metastatic	Platinum-based (carboplatin + paclitaxel)	Bevacizumab (mAb)	Atezolizumab	206	Not mentioned	Ad:36, Adsq:6, Sq:164	OS, PFS; ORR, DOR, safety
							204 (control)		Ad:43, Adsq:4, Sq:157	
X. Wu 2024 (11) (COMPASSION-16)	III	First-line	Persistent, recurrent, or metastatic	Platinum-based	Bevacizumab (optional per protocol) (mAb)	Cadonilimab (PD-1/CTLA-4)	133	Not mentioned	Not mentioned	PFS, OS; ORR, DOR, QOL, safety
							132 (control)			
D. Lorusso 2025 (20) (KEYNOTE-826)	III	First-line	Persistent, recurrent, or metastatic	Platinum-based (carboplatin/cisplatin + paclitaxel)	Bevacizumab (used in 63% of ICI arm) (mAb)	Pembrolizumab	196	CPS≥1%:74, CPS>10%:105, CPS<1%:21	Ad:37, Adsq:11, Sq:147	OS; PFS, DOR, safety
							193 (control)	CPS≥1%:41, CPS>10%:57, CPS<1%:14	Ad:59, Adsq:12, Sq:122	
Single-arm studies										
Qin Xu 2022 (21)	II	Later-line (PD-L1 positive)	Recurrent or metastatic	None	Anlotinib (TKI)	Sintilimab	42	CPS≥1%:42, CPS≥4:28, CPS<4:14	Ad:5, Sq:35, Adsq:2	ORR; PFS, DOR, safety
Lingfang Xia 2022 (22)	II	Later-line (squamous only)	Recurrent or metastatic	None	Famitinib (TKI)	Camrelizumab	33	CPS≥1%:10, CPS<1%:9, Unknown:14	Sq:33	ORR, PFS; OS, 12-month OS, DCR, safety
J. Zhu 2023 (23)	II	First-line	Persistent, recurrent, or metastatic	Platinum-based	Bevacizumab (mAb)	Tislelizumab	50	CPS≥1%:41, CPS<1%:6	Sq:46, Ad:4	PFS; OS, DCR, safety
Tse K.Y 2024 (24) (ALARICE)	II	Later-line (post-platinum)	Persistent or recurrent	None	Axitinib (TKI)	Avelumab	21	CPS≥1%:15	Sq:11	ORR, PFS; OS, DCR, DOR, QLQ, safety
Guo R 2024 (25)	II	First-line	Persistent, recurrent, or metastatic	None	Anlotinib (TKI)	AK105 (Penpulimab)	15	CPS≥1%:9, CPS<1%:1, Unknown:5	Sq:11	ORR, PFS; OS, DCR, DOR, safety
Wang D 2024 (26) (ALTER-GO-020)	II	First-line	Persistent, recurrent, or metastatic	None	Anlotinib (TKI)	Penpulimab	17	Not mentioned	Sq:11	ORR, PFS; OS, DCR, DOR, safety
Chunyan Lan 2024 (27) (CLAP)	II	Later-line	Advanced	None	Apatinib (TKI)	Camrelizumab	45	CPS≥1%:30, CPS<1%:10, Unknown:5	Ad:15, Sq:30	ORR, PFS; OS, DCR
Chen Li 2025 (28) (JS001-ISS-CO214)	II	First-line (refractory)	Recurrent or metastatic	Platinum-based	Bevacizumab (mAb)	Toripalimab	24	Not mentioned	Ad:5, Sq:19	ORR, PFS; OS, DCR, DOR, safety

### 3.3 Findings from randomized controlled trials (first−line treatment)

Three randomized controlled trials (RCTs) investigating first-line therapy for recurrent or metastatic cervical cancer (r/mCC) were eligible for inclusion. For the pooled analysis, we extracted data from pre-specified bevacizumab-containing subgroups in the KEYNOTE-826 and COMPASSION-16 trials, in which bevacizumab administration was not mandated. Additionally, we included the full dataset from the BEATcc trial, in which all enrolled patients received bevacizumab. Accordingly, the pooled study population comprised patients treated with immune checkpoint inhibitors (ICIs) plus bevacizumab and platinum-based chemotherapy, compared with those receiving bevacizumab plus chemotherapy alone.

#### 3.3.1 Overall survival

**Figure 2:**
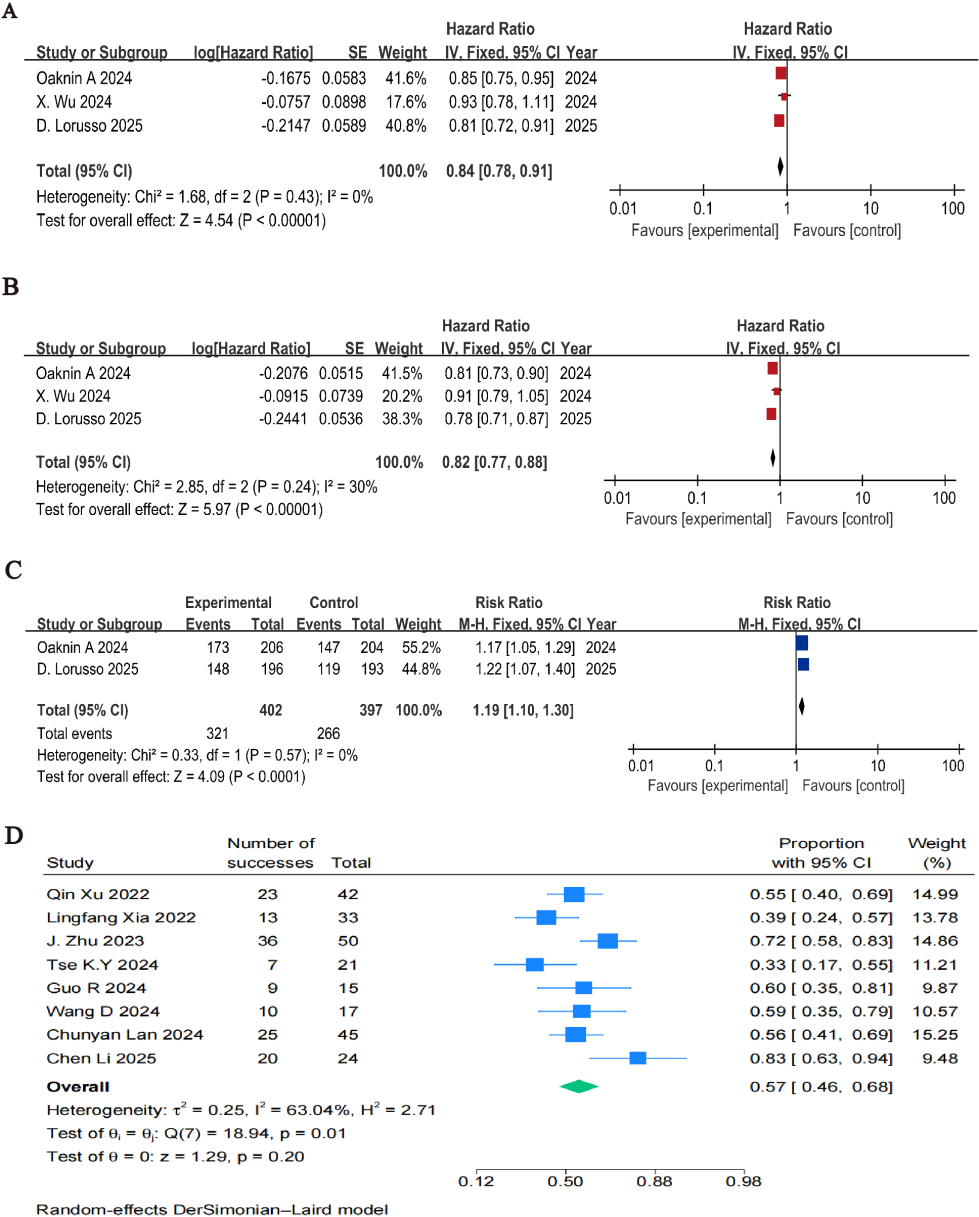
Forest plots of effectiveness outcomes for the included studies: **(A)** OS in RCTs; **(B)** PFS in RCTs; **(C)** ORR in RCTs; **(D)** ORR in single-arm studies.

The pooled analysis demonstrated a statistically significant improvement in OS for the combination therapy group compared to the control group. The HR for death was 0.84 (95% CI: 0.78–0.91, P < 0.001), indicating a 16% reduction in the risk of death (**Figure 2A**).

#### 3.3.2 Progression−free survival

The combination therapy also significantly prolonged PFS, with a pooled HR for progression or death of 0.82 (95% CI: 0.77–0.88, P < 0.001), corresponding to an 18% reduction in the risk of disease progression (**Figure 2B**).

#### 3.3.3 Objective response rate

ORR were reported in two RCTs. The ORR in the BEATcc study was 72% in the control arm and 84% in the atezolizumab-bevacizumab-chemotherapy arm. In the KEYNOTE-826 trial, the ORR was 50% in the placebo-chemotherapy arm and 75.5% in the pembrolizumab-containing arm. The combination group was favored by the pooled RR for objective response, which was 1.19 (95% CI: 1.10–1.30, P < 0.001) (**Figure 2C**).

#### 3.3.4 Safety

**Figure 3:**
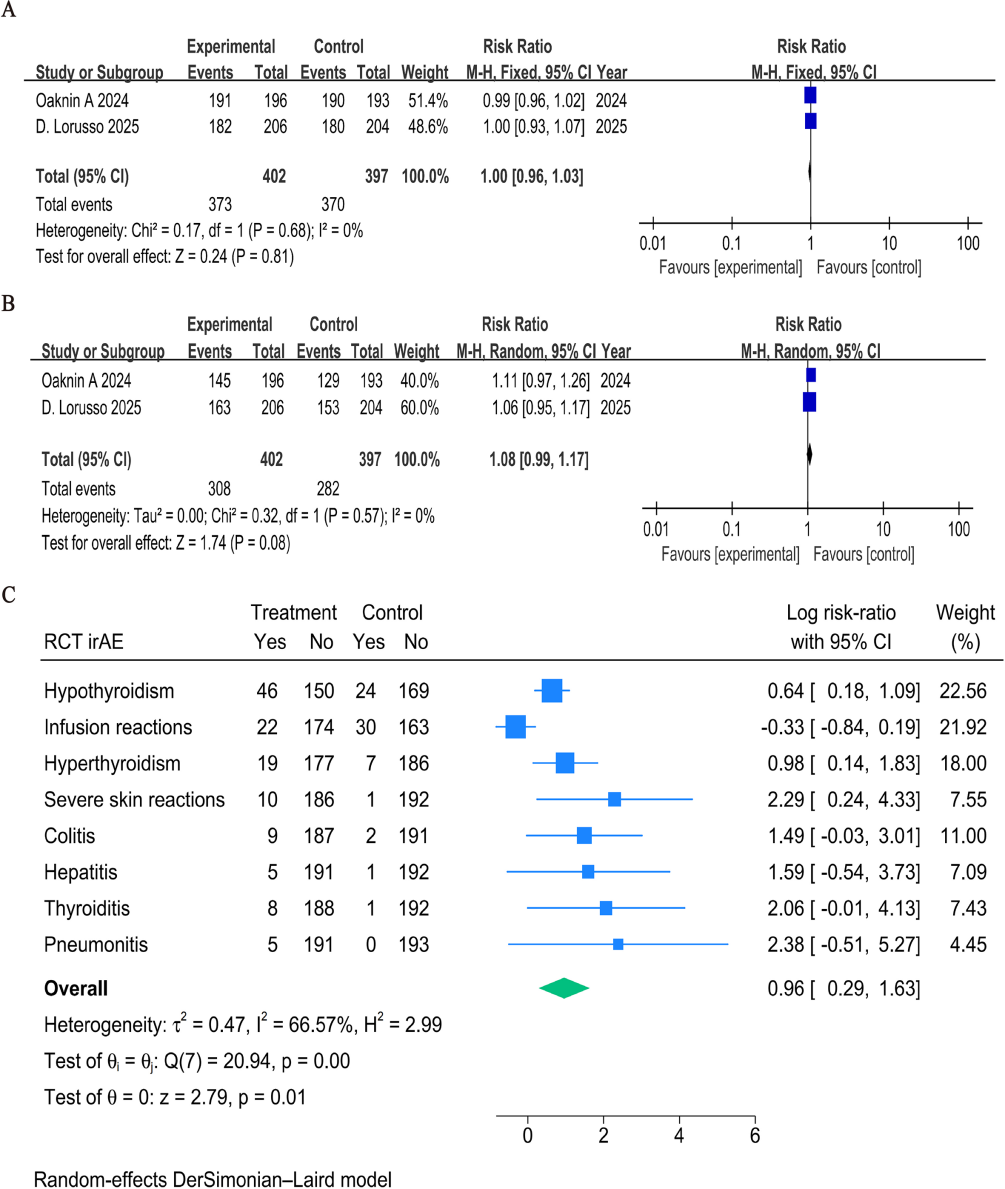
Forest plots of safety outcomes stratified by adverse events: **(A)** Adverse Events of Any Grade in RCTs; **(B)** ≥G3AEs in RCTs; **(C)** irAE in RCTs.

The combination and control groups had similar rates of any-grade treatment-related adverse events (TRAEs) (RR = 1.00, 95% CI: 0.96–1.03, P = 0.81; **Figure 3A**). The addition of ICIs did not significantly raise the incidence of grade ≥3 TRAEs (RR = 1.08, 95% CI: 0.99–1.17, P = 0.08; **Figure 3B**). However, the combination group experienced more immune-related adverse events (irAEs), especially hypothyroidism (RR = 1.89, 95% CI: 1.20–2.97) and hyperthyroidism (RR = 2.67, 95% CI: 1.15–6.21) (**Figure 3C**).

### 3.4 Findings from single−arm studies (later−line or mixed−line treatment)

Most patients with later-line or mixed-line progressed, recurring, or metastatic cervical cancer were included in eight single-arm trials. ICIs (camrelizumab, sintilimab, tislelizumab, avelumab, penpulimab, toripalimab) in combination with anti-angiogenic tyrosine kinase inhibitors (anlotinib, famitinib, apatinib, axitinib) or, in some studies, bevacizumab were among the many interventions. The descriptive results that follow should be viewed as exploratory.

#### 3.4.1 Overall survival

Two single−arm studies involving 66 patients reported median OS. Using a fixed−effects model (I² = 0.00%, P = 0.071), the pooled median OS was 20.5 months (95% CI: 10.66–30.52, P < 0.001) (**Supplementary Figure 1**).

#### 3.4.2 Progression−free survival

The median PFS was reported from five single-arm studies, including 149 individuals. Using a fixed-effects model (I² = 10.79%), the pooled median PFS was 10.26 months (95% CI: 7.85–12.67, P < 0.001) (**Supplementary Figure 2**).

#### 3.4.3 Objective response rate

The range of ORRs in single-arm studies was 33% to 83%. A random-effects model was used due to moderate heterogeneity (I² = 63.04%, P = 0.01), yielding a pooled ORR of 57% (95% CI: 0.46–0.68, P = 0.2) (**Figure 2D**). 85% (95% CI: 0.72–0.92, P = 0.00) was the pooled disease control rate (DCR) (**Supplementary Figure 3**).

#### 3.4.4 Safety

**Figure 4:**
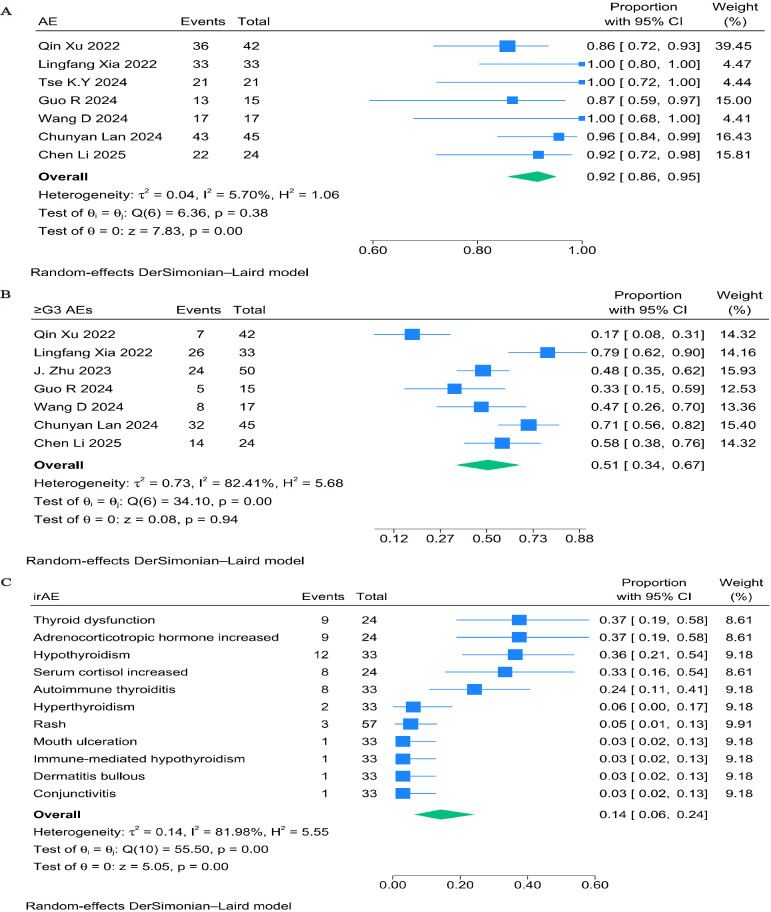
Forest plots of safety outcomes stratified by adverse events: **(A)** Adverse Events of Any Grade in single-arm studies; **(B)** ≥G3AEs in single-arm studies; **(C)** irAE in single-arm studies.

The majority of treatment-related adverse events in single-arm trials were grade 1 or 2. The combined incidence of TRAEs of any grade was 92% (95% CI: 86%–95%, P < 0.001; **Figure 4A**), while the incidence of TRAEs of grade ≥3 was 51% (95% CI: 34%–67%, P = 0.94; **Figure 4B**). Thyroid dysfunction (37%, 95% CI: 19%–58%), elevated adrenocorticotropic hormone (37%, 95% CI: 19%–58%), and hypothyroidism (36%, 95% CI: 21%–54%) were the most reported immune-related adverse events (**Figure 4C**).

#### 3.4.5 Exploratory subgroup analyses

**Figure 5:**
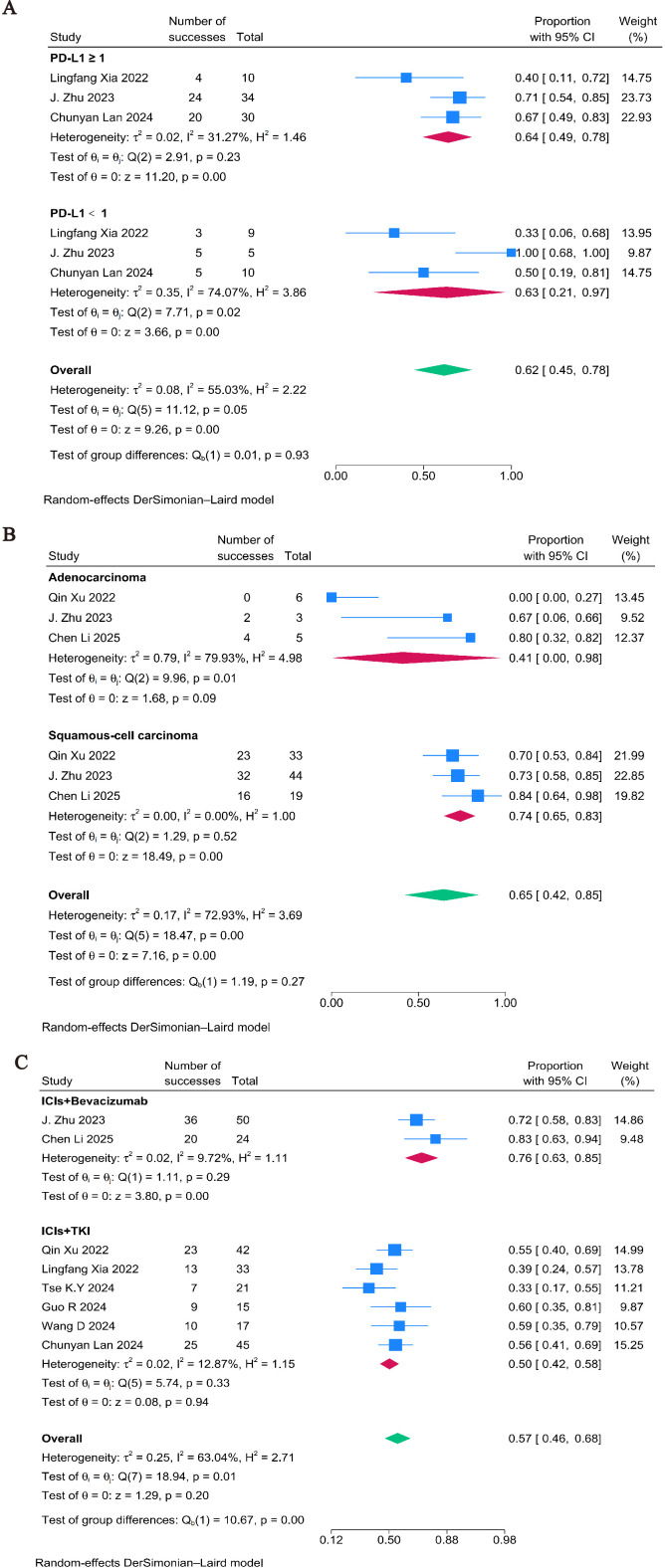
Forest plots of subgroup analysis categorized by clinical characteristics: **(A)** PD-L1 Expression Impact on ORR in Single-Arm Studies; **(B)** Histological Type Impact on ORR in Single- Arm Studies; **(C)** Treatment plan Impact on ORR in Single-Arm Studies.

We performed exploratory subgroup analyses using data from single−arm studies to generate hypotheses for future research. These analyses are limited by small sample sizes and a lack of comparative adjustment.PD−L1 expression status: The pooled ORR was 62% (95% CI: 0.45–0.78, P = 0.00) in patients with a PD−L1 combined positive score (CPS) ≥1% and 63% (95% CI: 0.45–0.78) in those with CPS <1%, suggesting a potential response signal even in PD−L1−negative tumors (**Figure 5A**). However, these estimates are imprecise and should be interpreted with caution. Histological subtype: For squamous cell carcinoma and adenocarcinoma, the pooled ORR was 74% (95% CI:0.65-0.83) and 41% (95% CI:0.00-0.98), respectively. Significant uncertainty is indicated by the latter’s large confidence ranges (**Figure 5B**). Type of anti-angiogenic agent: Tyrosine kinase inhibitors (TKIs) were linked to a lower pooled ORR (50%, 95% CI: 0.42–0.58) than combinations containing bevacizumab, a monoclonal antibody (76%, 95% CI: 0.63–0.85) (**Figure 5C**). This discrepancy needs to be validated in controlled trials because it may reflect different mechanisms or patient groups.

### 3.5 Sensitivity analysis and risk of bias

**Figure 6:**
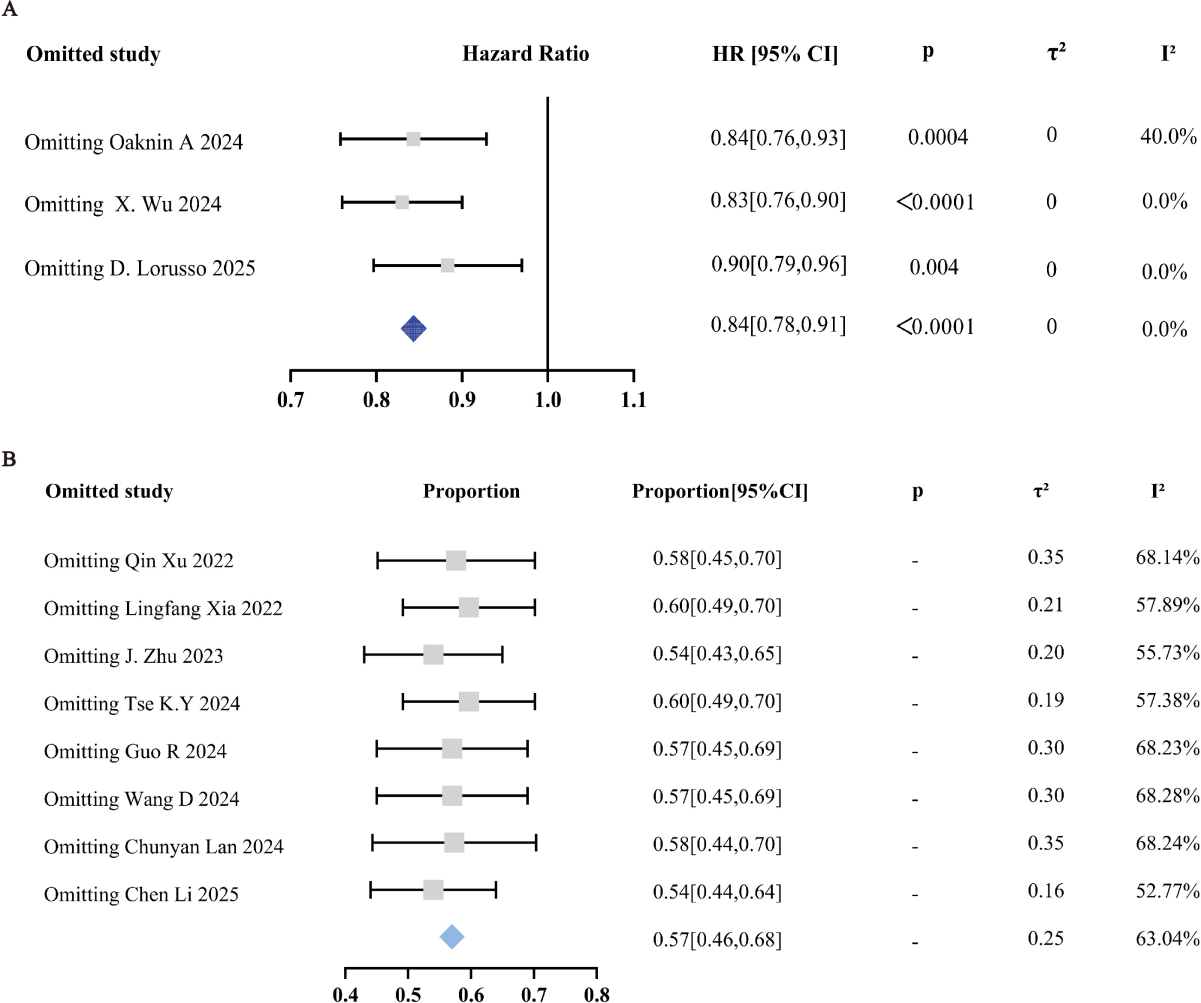
Sensitivity analysis of included studies: **(A)** RCTs; **(B)** single-arm studies.

Leave−one−out sensitivity analysis confirmed the robustness of the OS findings from RCTs, with HRs ranging from 0.83 to 0.90 and I² = 0% (**Figure 6A**). For the ORR in single−arm studies, the pooled ORR ranged from 54% to 60% with overlapping confidence intervals, and no single study dominated the outcome, although heterogeneity persisted (I² = 52.77%–68.28%) (**Figure 6B**). Risk of bias assessment classified the three RCTs as low risk (Cochrane RoB 2 tool). Among the eight single−arm studies, two were rated as low risk and six as moderate risk (ROBINS−I tool), indicating that study quality did not substantially affect the primary conclusion. Detailed risk−of−bias assessments are provided in **Supplementary Data 2**. To explore potential publication bias, we visually inspected funnel plots for the ORR of the eight single−arm studies; no gross asymmetry was observed (**Supplementary Figure 4**). Egger’s test (P = 0.587) and Begg’s test (P = 0.387) provided no statistical evidence of publication bias. For the three RCTs, formal publication bias tests are underpowered and were not performed. Publication bias remains a potential limitation given the small number of included studies (<10 per analysis), as acknowledged in the Discussion.

## 4 Discussion

Persistent high-risk human papillomavirus (HPV) infection is the leading cause of cervical cancer (CC). Despite this, the uptake of HPV vaccinations remains suboptimal, with only specific groups, such as women born after 1990, having had the chance to complete their vaccination schedules and achieve full immunization (29). Once CC advances to a more severe or recurrent stage, treatment options become increasingly limited (30). Recently, the widespread adoption of targeted therapies, including anti-angiogenic agents and antibody-drug conjugates (ADCs), alongside immune-based treatments like ICIs, has led to a growing body of successful clinical trial data. These developments signify a new era of precision medicine in the treatment of aCC and r/mCC (31).

The synergistic effects of combining ICIs with anti-angiogenic agents for aCC are supported by a strong biological rationale. Both therapeutic approaches target the TME. Anti-angiogenic agents, such as bevacizumab and anlotinib, inhibit the VEGF pathway, resulting in the normalization of tumor vasculature, which in turn enhances perfusion and oxygenation within the TME (32–34). These improvements facilitate better infiltration and functionality of cytotoxic T cells while also mitigating the VEGF-mediated suppression of dendritic cell maturation and reducing the expansion of regulatory T cell (Treg), thereby altering the immunosuppressive nature of the TME. Concurrently, ICIs help to reverse T cell exhaustion by blocking immune checkpoint pathways like PD-1/PD-L1. Collectively, these therapeutic mechanisms potentially establish a favourable anti-tumor immune cycle characterized by the sequence of “vessel normalization—immune cell infiltration—immune checkpoint disinhibition, “ working together to boost anti-tumor activity. Furthermore, research has shown that anti-angiogenic drugs can stimulate the formation of high endothelial venules (HEVs) (35). This leads to the hypothesis that tumor-associated high endothelial venules (TA-HEVs) are critical for lymphocyte infiltration into tumors. Increasing the density and maturation of TA-HEV endothelial cells (TA-HECs) may enhance CD8+ T cell infiltration and improve the effectiveness of ICIs (36, 37). However, additional clinical trials are needed to ascertain whether combination therapy or monotherapy with ICIs and anti-angiogenic agents can prolong overall survival for patients with aCC. Fortunately, several clinical trials are currently in progress to refine combination targeted- immunotherapy regimens for this disease, including notable studies such as NCT04982237, NCT05817214, and NCT0486588, which are exploring innovative combinations and strategies to enhance treatment outcomes (38–40).

This meta−analysis provides a systematic synthesis of existing evidence from RCTs and single−arm studies on the use of ICIs in combination with anti−angiogenic agents to treat aCC. Importantly, we explicitly stratified the evidence by clinical context: RCTs providing high−level evidence for first−line combinations (ICI + anti−angiogenic + chemotherapy), and single−arm studies offering exploratory insights into later−line ICI + TKI combinations. These contexts are not directly comparable and are interpreted separately. Our key findings, drawn from RCT data, reveal substantial survival benefits associated with combination therapy compared to standard treatments—primarily platinum−based chemotherapy with or without bevacizumab. The combined regimen led to a 16% reduction in the risk of death (OS HR = 0.84) and an 18% decrease in the risk of disease progression (PFS HR = 0.82), while also improving the objective response rate (ORR) by 19% (RR = 1.19). For patients ineligible for bevacizumab, chemotherapy combined with cadonilimab presents a significant alternative. These findings closely align with pivotal Phase III trial results, including KEYNOTE−826 and BEATcc, providing strong evidence supporting the combination as an important option in first−line treatment for aCC. While these comparisons are informative, we note that direct statistical comparisons across different trials are not justified, and differences in patient selection, concurrent therapies, and PD−L1 scoring methods may influence outcomes. Data from single−arm studies further suggest promising antitumor activity, particularly among refractory patient populations (pooled ORR: 57%). Notably, exploratory subgroup analyses suggest potential signals of response in traditionally less-responsive patient groups, including those with PD−L1−negative tumors (ORR: 63%) and adenocarcinoma histology (ORR: 41%). However, these findings are hypothesis−generating only due to the small number of events, wide confidence intervals, and lack of comparative adjustment in single−arm studies. Regarding safety, this analysis affirms that while ICIs are associated with a unique spectrum of irAEs, the combination of ICIs with anti−angiogenic agents demonstrated a safety profile that was considered clinically acceptable in the first-line trial setting, although it was not without significant toxicity. No significant increase in the incidence of overall AEs or grade ≥3 AEs was noted compared to the control group. However, the absolute rate of severe toxicity in single−arm studies was substantial (51% grade ≥3 TRAEs), which is clinically meaningful, especially for patients with prior pelvic radiation, impaired renal function, or poor performance status. The near−50% pooled incidence of grade ≥3 TRAEs in single−arm studies raises legitimate safety concerns, particularly for later−line, heavily pretreated patients receiving TKI−based regimens, emphasizing that the risk−benefit balance must be carefully weighed. Both RCTs and single-arm studies consistently report higher rates of AEs, emphasizing the need for vigilant monitoring, early detection, and proactive management of toxicities associated with combination therapy —especially irAEs, along with anti-angiogenic-related events such as hypertension, proteinuria, and bleeding. Careful patient selection and ongoing toxicity monitoring are crucial to ensuring treatment safety.

A thorough analysis of ORR data from single-arm studies reveals that PD-L1 expression status (CPS< 1) and pathologic subtype (adenocarcinoma vs. squamous cell carcinoma) do not act as absolute barriers to treatment response in combination therapy. These findings align with trends identified in specific subgroup analyses of RCTs indicating that the combination approach may reduce the biomarker dependency typically seen with ICIs used alone. Further subgroup analyses based on the type of anti-angiogenic agent suggest that combinations featuring bevacizumab, a monoclonal antibody, are associated with a higher ORR compared to those incorporating small-molecule tyrosine kinase inhibitors (TKIs). This observation calls for further validation in larger studies and mechanistic investigations to explore underlying differences, such as potential varying effects on the TME. Overall, these results highlight the necessity of developing biomarker-driven precision medicine strategies to optimize patient selection, expand the therapeutic window of combination regimens, and potentially reduce specific toxicity risks (41, 42).

This study has several limitations. First, the study design raises concerns regarding the potential for bias, particularly due to a high percentage of single-arm studies (72%). The lack of control groups increases the risk of selection bias in efficacy evaluations; for example, it may occur if patients with better performance status or those receiving earlier-line treatments are preferentially enrolled, leading to an overestimation of the treatment effect. Additionally, the limited number of studies included hinders a thorough assessment of publication bias. Second, beyond statistical heterogeneity, substantial clinical heterogeneity across included studies further limits the generalizability of our pooled estimates. Key sources include: (1) line of therapy (first−line vs later−line), (2) type of anti−angiogenic agent (monoclonal antibody vs. TKI), (3) concurrent chemotherapy use, and (4) varying PD−L1 thresholds. Therefore, our pooled estimates should not be interpreted as a single ‘class effect’. Third, the completeness of safety reporting is inadequate, with adverse events recorded inconsistently and incompletely across different studies. This lack of consistency significantly restricts the ability to conduct detailed statistical analyses of the safety profile of combination therapy, especially regarding specific rare or delayed-onset toxicities. Fourth, while the use of ROBINS−I for quality assessment of single−arm studies is the best available option, it is not ideal because this tool was designed for non−randomised comparative studies. This may have introduced some degree of bias in our quality assessment, and the results should be interpreted with this in mind. Publication bias for the ORR of single-arm studies was assessed using a funnel plot and Egger’s test (P = 0.587), neither of which suggested significant bias (**Supplementary Figure 4**). Formal testing was not feasible for the three RCTs due to the small number of studies. Finally, there is a widespread lack of reporting or incomplete data on essential biomarkers crucial for precision stratification, particularly concerning HPV status, microsatellite instability (MSI), and tumor mutation burden (TMB). This gap severely limits efforts to identify genetic markers that might predict the effects of combined treatments. As a result, there is an urgent need for well-designed RCTs with larger sample sizes and longer follow-up periods to confirm the clinical benefits of combining ICIs with anti-angiogenic agents.

## 5 Conclusion

Pooled analysis from first-line RCTs demonstrates that the integration of ICIs with anti-angiogenic agents and chemotherapy provides survival benefits for patients with persistent, recurrent, or metastatic cervical cancer, while ensuring a generally tolerable safety profile. However, the substantial rate of grade ≥3 treatment-related adverse events observed in single-arm studies (51%) underscores the need for careful patient selection and toxicity monitoring. Nevertheless, it is crucial to validate these findings through further RCTs. Future investigations should aim to clarify the positive feedback mechanisms that exist between ICIs and anti-angiogenic therapies.

## Data Availability

The original contributions presented in the study are included in the article/**Supplementary Material**. Further inquiries can be directed to the corresponding author.
