# Supplementary material for: Efficacy and safety of immune checkpoint inhibitors combined with antiangiogenic agents in advanced cervical cancer: a systematic review and meta-analysis
Source: Front Immunol. 2026 May 21;17:1747768. doi: 10.3389/fimmu.2026.1747768 (PMC13233532; doi:10.3389/fimmu.2026.1747768)
Supplement: Supplementary file 1 [file Supplementaryfile1.docx]

**Supplementary Date 1. Search Strategy for Databases**

1. PubMed

Search string

Subject heading: **Uterine Cervical Neoplasms;** **Immune Checkpoint Inhibitors;Angiogenesis Inhibitors**

**((("Uterine Cervical Neoplasms"[Mesh]) OR (((((((((((((((((((((((Uterine Cervical Neoplasms[Title/Abstract]) OR (Cervical Neoplasm, Uterine[Title/Abstract])) OR (Neoplasm, Uterine Cervical[Title/Abstract])) OR (Uterine Cervical Neoplasm[Title/Abstract])) OR (Neoplasms, Cervix[Title/Abstract])) OR (Cervix Neoplasm[Title/Abstract])) OR (Neoplasm, Cervix[Title/Abstract])) OR (Cervix Neoplasms[Title/Abstract])) OR (Cervical Neoplasms[Title/Abstract])) OR (Cervical Neoplasm[Title/Abstract])) OR (Neoplasms, Cervical[Title/Abstract])) OR (Cancer of the Uterine Cervix[Title/Abstract])) OR (Cancer of Cervix[Title/Abstract])) OR (Cancer of the Cervix[Title/Abstract])) OR (Cervix Cancer[Title/Abstract])) OR (Cancer, Cervix[Title/Abstract])) OR (Uterine Cervical Cancer[Title/Abstract])) OR (Cancer, Uterine Cervical[Title/Abstract])) OR (Cervical Cancer, Uterine[Title/Abstract])) OR (Uterine Cervical Cancers[Title/Abstract])) OR (Cervical Cancer[Title/Abstract])) OR (Cancer, Cervical[Title/Abstract])) OR (Cervical Cancers[Title/Abstract]))) AND (("Angiogenesis Inhibitors"[Mesh]) OR ((((((((((((((((((((((((((((((((((((((((((((((((((((((((((((((((((((((((Angiogenesis Inhibitors[Title/Abstract]) OR (Angiogenic Inhibitors[Title/Abstract])) OR (Angiostatic Agents[Title/Abstract])) OR (Agents, Angiostatic[Title/Abstract])) OR (Antagonists, Angiogenic[Title/Abstract])) OR (Anti-Angiogenetic Agents[Title/Abstract])) OR (Agents, Anti-Angiogenetic[Title/Abstract])) OR (Anti Angiogenetic Agents[Title/Abstract])) OR (Anti-Angiogenic Drugs[Title/Abstract])) OR (Anti Angiogenic Drugs[Title/Abstract])) OR (Drugs, Anti-Angiogenic[Title/Abstract])) OR (Antiangiogenic Agents[Title/Abstract])) OR (Agents, Antiangiogenic[Title/Abstract])) OR (Inhibitors, Angiogenesis[Title/Abstract])) OR (Inhibitors, Angiogenetic[Title/Abstract])) OR (Inhibitors, Angiogenic[Title/Abstract])) OR (Inhibitors, Neovascularization[Title/Abstract])) OR (Neovascularization Inhibitors[Title/Abstract])) OR (Anti-Angiogenic Drug[Title/Abstract])) OR (Anti Angiogenic Drug[Title/Abstract])) OR (Drug, Anti-Angiogenic[Title/Abstract])) OR (Neovascularization Inhibitor[Title/Abstract])) OR (Inhibitor, Neovascularization[Title/Abstract])) OR (Antiangiogenic Agent[Title/Abstract])) OR (Agent, Antiangiogenic[Title/Abstract])) OR (Angiogenesis Inhibitor[Title/Abstract])) OR (Inhibitor, Angiogenesis[Title/Abstract])) OR (Angiogenetic Antagonist[Title/Abstract])) OR (Antagonist, Angiogenetic[Title/Abstract])) OR (Angiogenetic Antagonists[Title/Abstract])) OR (Antagonists, Angiogenetic[Title/Abstract])) OR (Angiogenetic Inhibitor[Title/Abstract])) OR (Inhibitor, Angiogenetic[Title/Abstract])) OR (Angiogenetic Inhibitors[Title/Abstract])) OR (Angiogenic Antagonists[Title/Abstract])) OR (Angiogenic Antagonist[Title/Abstract])) OR (Antagonist, Angiogenic[Title/Abstract])) OR (Angiogenic Inhibitor[Title/Abstract])) OR (Inhibitor, Angiogenic[Title/Abstract])) OR (Angiostatic Agent[Title/Abstract])) OR (Agent, Angiostatic[Title/Abstract])) OR (Anti-Angiogenetic Agent[Title/Abstract])) OR (Agent, Anti-Angiogenetic[Title/Abstract])) OR (Anti Angiogenetic Agent[Title/Abstract])) OR (Angiogenesis Factor Inhibitors[Title/Abstract])) OR (Factor Inhibitors, Angiogenesis[Title/Abstract])) OR (Inhibitors, Angiogenesis Factor[Title/Abstract])) OR (Angiogenesis Factor Inhibitor[Title/Abstract])) OR (Factor Inhibitor, Angiogenesis[Title/Abstract])) OR (Inhibitor, Angiogenesis Factor[Title/Abstract])) OR (Anti-Angiogenesis Effect[Title/Abstract])) OR (Anti Angiogenesis Effect[Title/Abstract])) OR (Effect, Anti-Angiogenesis[Title/Abstract])) OR (Anti-Angiogenesis Effects[Title/Abstract])) OR (Anti Angiogenesis Effects[Title/Abstract])) OR (Effects, Anti-Angiogenesis[Title/Abstract])) OR (Antiangiogenesis Effect[Title/Abstract])) OR (Effect, Antiangiogenesis[Title/Abstract])) OR (Antiangiogenesis Effects[Title/Abstract])) OR (Effects, Antiangiogenesis[Title/Abstract])) OR (bevacizumab[Title/Abstract])) OR (apatinib[Title/Abstract])) OR (VEGF[Title/Abstract])) OR (VEGFR[Title/Abstract])) OR (PDGFR[Title/Abstract])) OR (Lenvatinib[Title/Abstract])) OR (Avastin[Title/Abstract])) OR (Sorafenib[Title/Abstract])) OR (Sunitinib[Title/Abstract])) OR (Anlotinib[Title/Abstract])) OR (Regorafenib[Title/Abstract])) OR (Pazopanib[Title/Abstract])))) AND (("Immune Checkpoint Inhibitors"[Mesh]) OR (((((((((((((((((((((((((((((((((((((((((Immune Checkpoint Inhibitors[Title/Abstract]) OR (ICIs[Title/Abstract])) OR (Checkpoint Inhibitors, Immune[Title/Abstract])) OR (Immune Checkpoint Blockers[Title/Abstract])) OR (Checkpoint Blockers, Immune[Title/Abstract])) OR (Immune Checkpoint Inhibitor[Title/Abstract])) OR (Checkpoint Inhibitor, Immune[Title/Abstract])) OR (CTLA-4 Inhibitors[Title/Abstract])) OR (CTLA 4 Inhibitors[Title/Abstract])) OR (Cytotoxic T-Lymphocyte-Associated Protein 4 Inhibitors[Title/Abstract])) OR (Cytotoxic T Lymphocyte Associated Protein 4 Inhibitors[Title/Abstract])) OR (PD-1 Inhibitors[Title/Abstract])) OR (PD 1 Inhibitors[Title/Abstract])) OR (Programmed Cell Death Protein 1 Inhibitors[Title/Abstract])) OR (Inhibitor, PD-1[Title/Abstract])) OR (Immune Checkpoint Blockade[Title/Abstract])) OR (Checkpoint Blockade, Immune[Title/Abstract])) OR (Immune Checkpoint Inhibition[Title/Abstract])) OR (Checkpoint Inhibition, Immune[Title/Abstract])) OR (PD-L1 Inhibitors[Title/Abstract])) OR (PD L1 Inhibitors[Title/Abstract])) OR (Programmed Death-Ligand 1 Inhibitors[Title/Abstract])) OR (Programmed Death Ligand 1 Inhibitors[Title/Abstract])) OR (Blockade, PD-1-PD-L1[Title/Abstract])) OR (PD 1 PD L1 Blockade[Title/Abstract])) OR (Pembrolizumab[Title/Abstract])) OR (Nivolumab[Title/Abstract])) OR (Atezolizumab[Title/Abstract])) OR (Durvalumab[Title/Abstract])) OR (Cemiplimab[Title/Abstract])) OR (Camrelizumab[Title/Abstract])) OR (Sintilimab[Title/Abstract])) OR (Tislelizumab[Title/Abstract])) OR (Toripalimab[Title/Abstract])) OR (Avelumab[Title/Abstract])) OR (Tremelimumab[Title/Abstract])) OR (Ipilimumab[Title/Abstract])) OR (Dostarlimab[Title/Abstract])) OR (Balstilimab[Title/Abstract])) OR (Camrelizumab[Title/Abstract])) OR (Cadonilimab[Title/Abstract])))**

**Search returned 136 articles;**

2.Embase

Search string

1. **'uterine cervix tumor'/exp**
2. **'cervical neoplasia':ab,ti OR 'cervical neoplasm'/exp OR 'cervical tumor':ab,ti OR 'cervical tumorigenesis':ab,ti OR 'cervical tumour':ab,ti OR 'cervix neoplasia':ab,ti OR 'cervix neoplasm':ab,ti OR 'cervix neoplasms':ab,ti OR 'cervix tumor':ab,ti OR 'cervix tumorigenesis':ab,ti OR 'cervix tumour':ab,ti OR 'cervix uteri tumor':ab,ti OR 'neoplasia of the cervix':ab,ti OR 'neoplasm of the cervix':ab,ti OR 'neoplastic cervical':ab,ti OR 'neoplastic cervix':ab,ti OR 'tumor of the cervix':ab,ti OR 'tumor of the uterine cervix':ab,ti OR 'tumour of the cervix':ab,ti OR 'tumour of the uterine cervix':ab,ti OR 'uterine cervical neoplasia':ab,ti OR 'uterine cervical neoplasm':ab,ti OR 'uterine cervical neoplasms':ab,ti OR 'uterine cervical tumor':ab,ti OR 'uterine cervix neoplasia':ab,ti OR 'uterine cervix neoplasm':ab,ti OR 'uterine cervix tumour':ab,ti OR 'uterine cervix tumor':ab,ti**
3. **#1 OR #2**
4. **'****immune checkpoint inhibitor'/exp**
5. **'immune checkpoint inhibitor'/exp OR 'immune checkpoint inhibitor':ab,ti OR 'pd 1 inhibitor':ab,ti OR 'immune checkpoint blockade':ab,ti OR 'immune checkpoint inhibition':ab,ti OR 'pd l1 inhibitor':ab,ti OR 'programmed death ligand 1 inhibitor':ab,ti OR 'programmed cell death protein 1 inhibitor':ab,ti OR 'pembrolizumab':ab,ti OR 'nivolumab':ab,ti OR 'atezolizumab':ab,ti OR 'durvalumab':ab,ti OR 'cemiplimab':ab,ti OR 'ipilimumab':ab,ti OR 'camrelizumab':ab,ti OR 'cadonilimab':ab,ti**
6. **#4 OR #5**
7. **'angiogenesis inhibitor'/exp**
8. **'angiogenesis inhibitor':ab,ti OR 'angiostatic agents':ab,ti OR 'agents, angiostatic':ab,ti OR 'anti-angiogenic drugs':ab,ti OR 'anti angiogenic drugs':ab,ti OR 'inhibitors, angiogenetic':ab,ti OR bevacizumab:ab,ti OR lenvatinib:ab,ti OR 'bevacizumab':ab,ti OR sorafenib:ab,ti OR sunitinib:ab,ti OR apatinib:ab,ti OR cabozantinib:ab,ti OR anlotinib:ab,ti**
9. **#7 OR #8**
10. **#3 AND #6 AND #9**

**Search returned 691 articles;**

3. Web of Science

Search string

1. **((((((((((((((((((((((TS=(Uterine Cervical Neoplasms)) OR TS=(Cervical Neoplasm, Uterine)) OR TS=(Neoplasm, Uterine Cervical)) OR TS=(Uterine Cervical Neoplasm)) OR TS=(Neoplasms, Cervix)) OR TS=(Cervix Neoplasm)) OR TS=(Neoplasm, Cervix)) OR TS=(Cervix Neoplasms)) OR TS=( Cervical Neoplasms)) OR TS=(Cervical Neoplasm)) OR TS=(Neoplasms, Cervical)) OR TS=( Cancer of the Uterine Cervix)) OR TS=(Cancer of Cervix)) OR TS=( Cancer of the Cervix)) OR TS=(Cervix Cancer)) OR TS=(Cancer, Cervix)) OR TS=(Uterine Cervical Cancer)) OR TS=( Cancer, Uterine Cervical)) OR TS=(Cervical Cancer, Uterine)) OR TS=(Uterine Cervical Cancers)) OR TS=(Cervical Cancer)) OR TS=( Cancer, Cervical)) OR TS=( Cervical Cancers) and Preprint Citation Index (Exclude – Database)**
2. **((((((((((((((((((((((((((((((((((((((((TS=(Immune Checkpoint Inhibitors)) OR TS=(ICIs)) OR TS=(Checkpoint Inhibitors, Immune)) OR TS=(Immune Checkpoint Blockers)) OR TS=(Checkpoint Blockers, Immune)) OR TS=(Immune Checkpoint Inhibitor)) OR TS=(Checkpoint Inhibitor, Immune)) OR TS=(CTLA-4 Inhibitors)) OR TS=(CTLA 4 Inhibitors)) OR TS=(Cytotoxic T-Lymphocyte-Associated Protein 4 Inhibitors)) OR TS=(Cytotoxic T Lymphocyte Associated Protein 4 Inhibitors)) OR TS=(PD-1 Inhibitors)) OR TS=(PD 1 Inhibitors)) OR TS=(Programmed Cell Death Protein 1 Inhibitors)) OR TS=(Inhibitor, PD-1)) OR TS=(Immune Checkpoint Blockade)) OR TS=(Checkpoint Blockade, Immune)) OR TS=(Immune Checkpoint Inhibition)) OR TS=(Checkpoint Inhibition, Immune)) OR TS=(PD-L1 Inhibitors)) OR TS=(PD L1 Inhibitors)) OR TS=(Programmed Death-Ligand 1 Inhibitors)) OR TS=(Programmed Death Ligand 1 Inhibitors)) OR TS=(Blockade, PD-1-PD-L1)) OR TS=(PD 1 PD L1 Blockade)) OR TS=(Pembrolizumab)) OR TS=(Nivolumab)) OR TS=(Atezolizumab)) OR TS=(Durvalumab)) OR TS=(Cemiplimab)) OR TS=(Camrelizumab)) OR TS=(Sintilimab)) OR TS=(Tislelizumab)) OR TS=(Toripalimab)) OR TS=(Avelumab)) OR TS=(Tremelimumab)) OR TS=(Ipilimumab)) OR TS=(Dostarlimab)) OR TS=(Balstilimab)) OR TS=(Camrelizumab)) OR TS=(Cadonilimab) and Preprint Citation Index (Exclude – Database)**
3. **(((((((((((((((((((((((((((((((((((((((((((((((((((((((((((((((((((((((TS=(Angiogenesis Inhibitors)) OR TS=(Angiogenic Inhibitors)) OR TS=(Angiostatic Agents)) OR TS=(Agents, Angiostatic)) OR TS=(Antagonists, Angiogenic)) OR TS=(Anti-Angiogenetic Agents)) OR TS=(Agents, Anti-Angiogenetic)) OR TS=(Anti Angiogenetic Agents)) OR TS=(Anti-Angiogenic Drugs)) OR TS=(Anti Angiogenic Drugs)) OR TS=(Drugs, Anti-Angiogenic)) OR TS=(Antiangiogenic Agents)) OR TS=(Agents, Antiangiogenic)) OR TS=(Inhibitors, Angiogenesis)) OR TS=(Inhibitors, Angiogenetic)) OR TS=(Inhibitors, Angiogenic)) OR TS=(Inhibitors, Neovascularization)) OR TS=(Neovascularization Inhibitors)) OR TS=(Anti-Angiogenic Drug)) OR TS=(Anti Angiogenic Drug)) OR TS=(Drug, Anti-Angiogenic)) OR TS=(Neovascularization Inhibitor)) OR TS=(Inhibitor, Neovascularization)) OR TS=(Antiangiogenic Agent)) OR TS=(Agent, Antiangiogenic)) OR TS=(Angiogenesis Inhibitor)) OR TS=(Inhibitor, Angiogenesis)) OR TS=(Angiogenetic Antagonist)) OR TS=(Antagonist, Angiogenetic)) OR TS=(Angiogenetic Antagonists)) OR TS=(Antagonists, Angiogenetic)) OR TS=(Angiogenetic Inhibitor)) OR TS=(Inhibitor, Angiogenetic)) OR TS=(Angiogenetic Inhibitors)) OR TS=(Angiogenic Antagonists)) OR TS=(Angiogenic Antagonist)) OR TS=(Antagonist, Angiogenic)) OR TS=(Angiogenic Inhibitor)) OR TS=(Inhibitor, Angiogenic)) OR TS=(Angiostatic Agent)) OR TS=(Agent, Angiostatic)) OR TS=(Anti-Angiogenetic Agent)) OR TS=(Agent, Anti-Angiogenetic)) OR TS=(Anti Angiogenetic Agent)) OR TS=(Angiogenesis Factor Inhibitors)) OR TS=(Factor Inhibitors, Angiogenesis)) OR TS=(Inhibitors, Angiogenesis Factor)) OR TS=(Angiogenesis Factor Inhibitor)) OR TS=(Factor Inhibitor, Angiogenesis)) OR TS=(Inhibitor, Angiogenesis Factor)) OR TS=(Anti-Angiogenesis Effect)) OR TS=(Anti Angiogenesis Effect)) OR TS=(Effect, Anti-Angiogenesis)) OR TS=(Anti-Angiogenesis Effects)) OR TS=(Anti Angiogenesis Effects)) OR TS=(Effects, Anti-Angiogenesis)) OR TS=(Antiangiogenesis Effect)) OR TS=(Effect, Antiangiogenesis)) OR TS=(Antiangiogenesis Effects)) OR TS=(Effects, Antiangiogenesis)) OR TS=(bevacizumab)) OR TS=(apatinib)) OR TS=(VEGF)) OR TS=(VEGFR)) OR TS=(PDGFR)) OR TS=(Lenvatinib)) OR TS=(Avastin)) OR TS=(Sorafenib)) OR TS=(Sunitinib)) OR TS=(Anlotinib)) OR TS=(Regorafenib)) OR TS=(Pazopanib) and Preprint Citation Index (Exclude – Database)**
4. **#3 AND #2 AND #1 and Preprint Citation Index (Exclude – Database)**

**Search returned 303 articles;**

4. Cochrane

Search string

Subject heading: Uterine Cervical Neoplasms; Immune Checkpoint Inhibitors;**Angiogenesis Inhibitors**

1. **MeSH descriptor: [Uterine Cervical Neoplasms] explode all trees**
2. **Neoplasm or Uterine Cervical or Cervical Neoplasm, Uterine or Uterine Cervical Neoplasm or Cervix Neoplasms or Neoplasms, Cervical or Cervix Neoplasm or Neoplasm, Cervix or Neoplasms, Cervix or Cervical Neoplasms or Cervical Neoplasm or Cervical Cancers or Cancer of Cervix or Uterine Cervical Cancer or Cervix Cancer or Cancer, Cervix or Cancer, Uterine Cervical or Uterine Cervical Cancers or Cancer of the Uterine Cervix or Cervical Cancer or Cervical Cancer, Uterine or Cancer, Cervical or Cancer of the Cervix**
3. **#1 or #2**
4. **MeSH descriptor: [Immune Checkpoint Inhibitors] explode all trees**
5. **PD L1 Inhibitors or PD L1 Inhibitor or Programmed Death-Ligand 1 Inhibitors or Programmed Death Ligand 1 Inhibitors or PD 1 PD L1 Blockade or Immune Checkpoint Blockers or Immune Checkpoint Inhibitor or Immune Checkpoint Inhibitors or CTLA-4 Inhibitor or Cytotoxic T-Lymphocyte-Associated Protein 4 Inhibitor or Cytotoxic T-Lymphocyte-Associated Protein 4 Inhibitors or CTLA-4 Inhibitors or Cytotoxic T Lymphocyte Associated Protein 4 Inhibitor or Cytotoxic T Lymphocyte Associated Protein 4 Inhibitors or PD 1 Inhibitors or PD 1 Inhibitor or Programmed Cell Death Protein 1 Inhibitor or Programmed Cell Death Protein 1 Inhibitors or Immune Checkpoint Inhibition or Immune Checkpoint Blockade**
6. **#4 or #5**
7. **MeSH descriptor: [Angiogenesis Inhibitors] explode all trees**
8. **Angiogenesis Factor Inhibitors or Factor Inhibitors, Angiogenesis or Inhibitors, Angiogenesis Factor or Angiogenesis Factor Inhibitor or Factor Inhibitor, Angiogenesis or Inhibitor, Angiogenesis Factor or Anti Angiogenesis Effect or Anti Angiogenesis Effects or Effect, Antiangiogenesis or Anti-Angiogenesis Effects or Antiangiogenesis Effects or Effects, Anti-Angiogenesis or Antiangiogenesis Effect or Effect, Anti-Angiogenesis or Effects, Antiangiogenesis or Anti-Angiogenesis Effect or Angiostatic Agent or Anti-Angiogenetic Agents or Agents, Angiostatic or Anti Angiogenic Drug or Angiogenetic Inhibitors or Antiangiogenic Agents or Inhibitors, Neovascularization**
9. **#7 or #8**
10. **#3 and #6 and #9**

**Search returned 66 article**

5. SCOPUS

Search string

**( TITLE-ABS-KEY ( "Uterine Cervical Neoplasms" OR "Cervical Neoplasm, Uterine" OR "Neoplasm, Uterine Cervical" OR "Uterine Cervical Neoplasm" OR "Neoplasms, Cervix" OR "Cervix Neoplasm" OR "Neoplasm, Cervix" OR "Cervix Neoplasms" OR "Cervical Neoplasms" OR "Cervical Neoplasm" OR "Neoplasms, Cervical" OR "Cancer of the Uterine Cervix" OR "Cancer of Cervix" OR "Cancer of the Cervix" OR "Cervix Cancer" OR "Cancer, Cervix" OR "Uterine Cervical Cancer" OR "Cancer, Uterine Cervical" OR "Cervical Cancer, Uterine" OR "Uterine Cervical Cancers" OR "Cervical Cancer" OR "Cancer, Cervical" OR "Cervical Cancers" ) AND TITLE-ABS-KEY ( "Angiogenesis Inhibitors" OR "Angiogenic Inhibitors" OR "Angiostatic Agents" OR "Agents, Angiostatic" OR "Antagonists, Angiogenic" OR "Inhibitors, Neovascularization" OR "Neovascularization Inhibitors" OR "Anti-Angiogenic Drug" OR "Angiogenic Antagonist" OR "Angiostatic Agent" OR "Agent, Angiostatic" OR "Anti-Angiogenetic Agent" OR "Agent, Anti-Angiogenetic" OR "Anti Angiogenetic Agent" OR "Angiogenesis Factor Inhibitors" OR "Factor Inhibitors, Angiogenesis" OR "Inhibitors, Angiogenesis Factor" OR "Angiogenesis Factor Inhibitor" OR "Factor Inhibitor, Angiogenesis" OR "Inhibitor, Angiogenesis Factor" OR "Anti-Angiogenesis Effect" OR "Anti Angiogenesis Effect" OR "Effect, Anti-Angiogenesis" OR "Anti-Angiogenesis Effects" OR "Anti Angiogenesis Effects" OR "Effects, Anti-Angiogenesis" OR "Antiangiogenesis Effect" OR "Effect, Antiangiogenesis" OR "Antiangiogenesis Effects" OR "Effects, Antiangiogenesis" OR "bevacizumab" OR "apatinib" OR "VEGF" OR "VEGFR" OR "PDGFR" OR "Lenvatinib" OR "Avastin" OR "Sorafenib" OR "Sunitinib" OR "Anlotinib" OR "Regorafenib" OR "Pazopanib" ) AND TITLE-ABS-KEY ( "Immune Checkpoint Inhibitors" OR "Immune Checkpoint Blockers" OR "Checkpoint Blockers, Immune" OR "Immune Checkpoint Inhibitor" OR "Checkpoint Inhibitor, Immune" OR "CTLA-4 Inhibitors" OR "CTLA 4 Inhibitors" OR "Cytotoxic T-Lymphocyte-Associated Protein 4 Inhibitors" OR "Cytotoxic T Lymphocyte Associated Protein 4 Inhibitors" OR "Cytotoxic T-Lymphocyte-Associated Protein 4 Inhibitor" OR "Cytotoxic T Lymphocyte Associated Protein 4 Inhibitor" OR "CTLA-4 Inhibitor" OR "CTLA 4 Inhibitor" OR "PD-1 Inhibitors" OR "PD 1 Inhibitors" OR "Programmed Cell Death Protein 1 Inhibitor" OR "Programmed Cell Death Protein 1 Inhibitors" "PD-1 Inhibitor" OR "Inhibitor, PD-1" OR "PD 1 Inhibitor" OR "Immune Checkpoint Blockade" OR "Checkpoint Blockade, Immune" OR "Immune Checkpoint Inhibition" OR "Checkpoint Inhibition, Immune" OR "PD-L1 Inhibitors" OR "PD L1 Inhibitors" OR "Programmed Death-Ligand 1 Inhibitors" OR "Programmed Death Ligand 1 Inhibitors" OR "PD-L1 Inhibitor" OR "PD L1 Inhibitor" OR "PD-1-PD-L1 Blockade" OR "Blockade, PD-1-PD-L1" OR "PD 1 PD L1 Blockade" OR "Pembrolizumab" OR "Nivolumab" OR "Atezolizumab" OR "Durvalumab" OR "Cemiplimab" OR "Camrelizumab" OR "Sintilimab" OR "Tislelizumab" OR "Toripalimab" OR "Avelumab" OR "Tremelimumab" OR "Ipilimumab" OR "Dostarlimab" OR "Balstilimab" OR "Camrelizumab" OR "Cadonilimab" ) )**

**Search returned 210 article**
