# Supplementary material for: Efficacy and safety of immune checkpoint inhibitors combined with antiangiogenic agents in advanced cervical cancer: a systematic review and meta-analysis
Source: Front Immunol. 2026 May 21;17:1747768. doi: 10.3389/fimmu.2026.1747768 (PMC13233532; doi:10.3389/fimmu.2026.1747768)

**Supplementary Data 2. Risk of bias assessment**

**1.Cochrane** Assessment Form

**
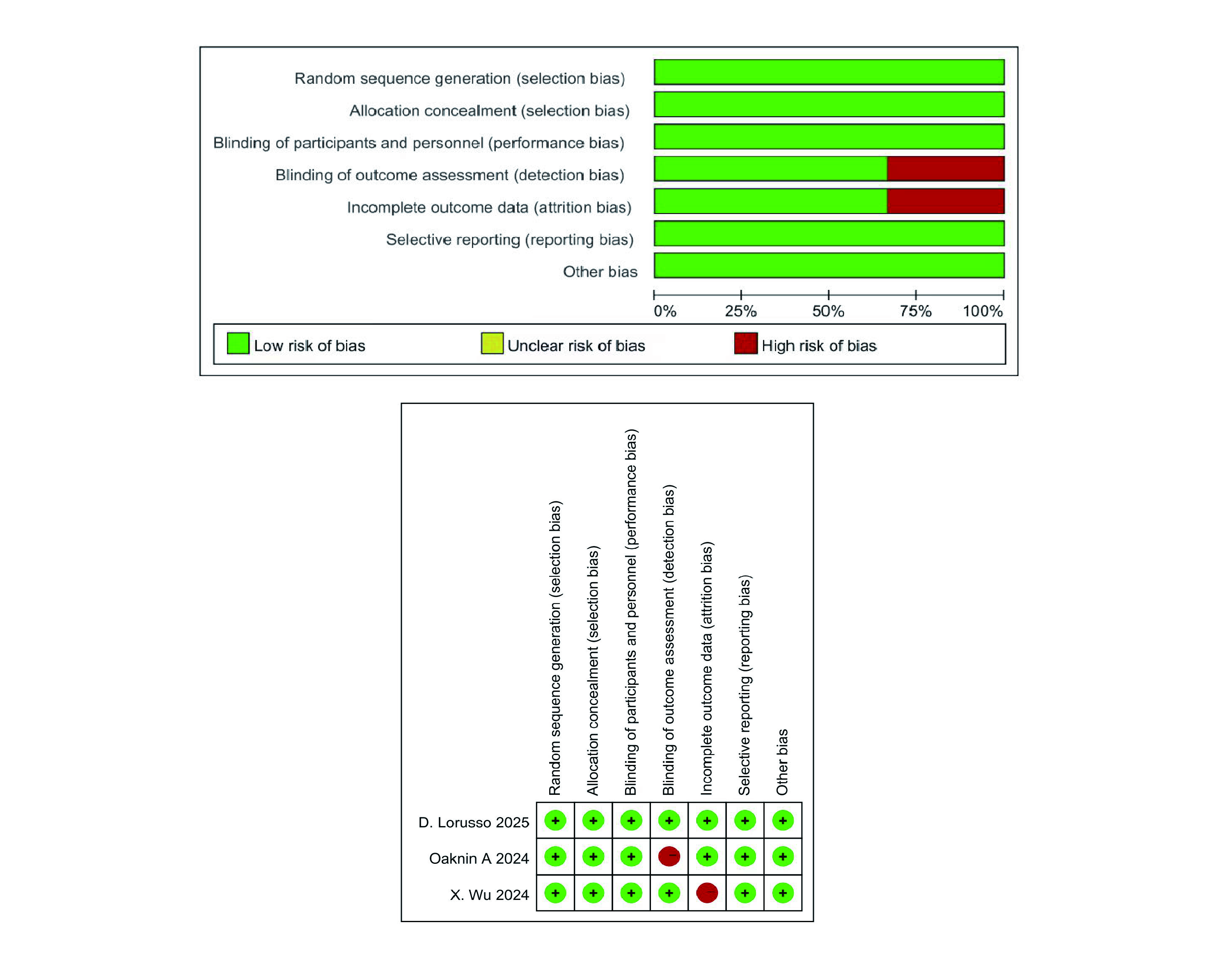
**

2.Risk of bias for the 11 included publications (single arm studies), based on the ROBINS-I tool (low, moderate, serious, critical)


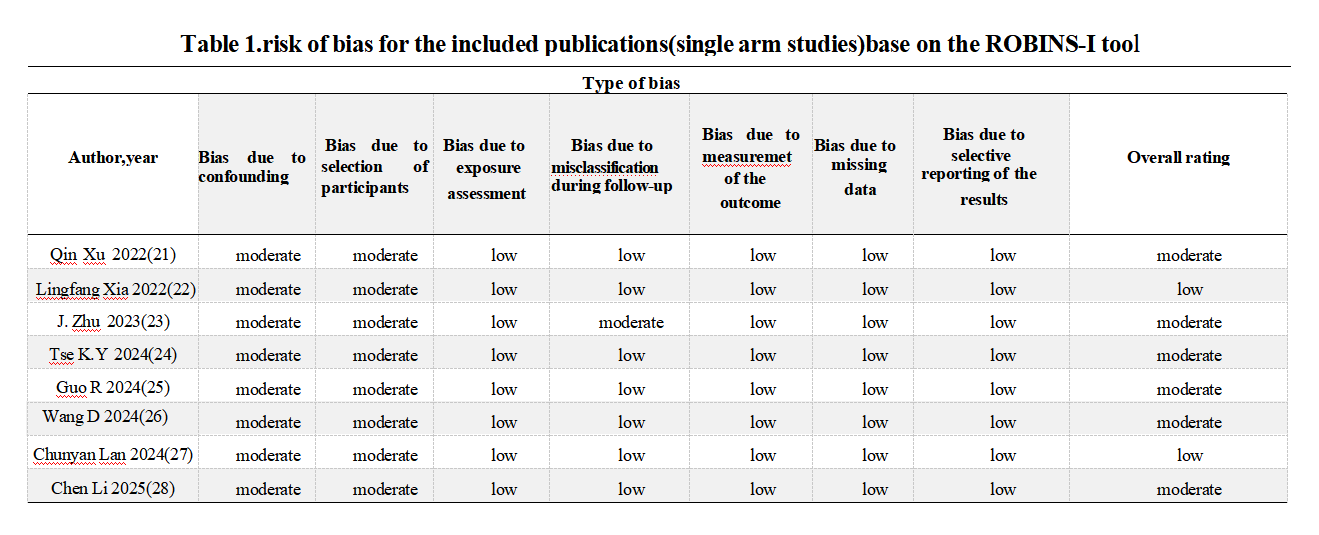

Supplement: Supplementary file 2 [file Supplementaryfile2.docx]
