# Supplementary material for: Efficacy and safety of immune checkpoint inhibitors combined with antiangiogenic agents in advanced cervical cancer: a systematic review and meta-analysis
Source: Front Immunol. 2026 May 21;17:1747768. doi: 10.3389/fimmu.2026.1747768 (PMC13233532; doi:10.3389/fimmu.2026.1747768)

## Supplementary Figures


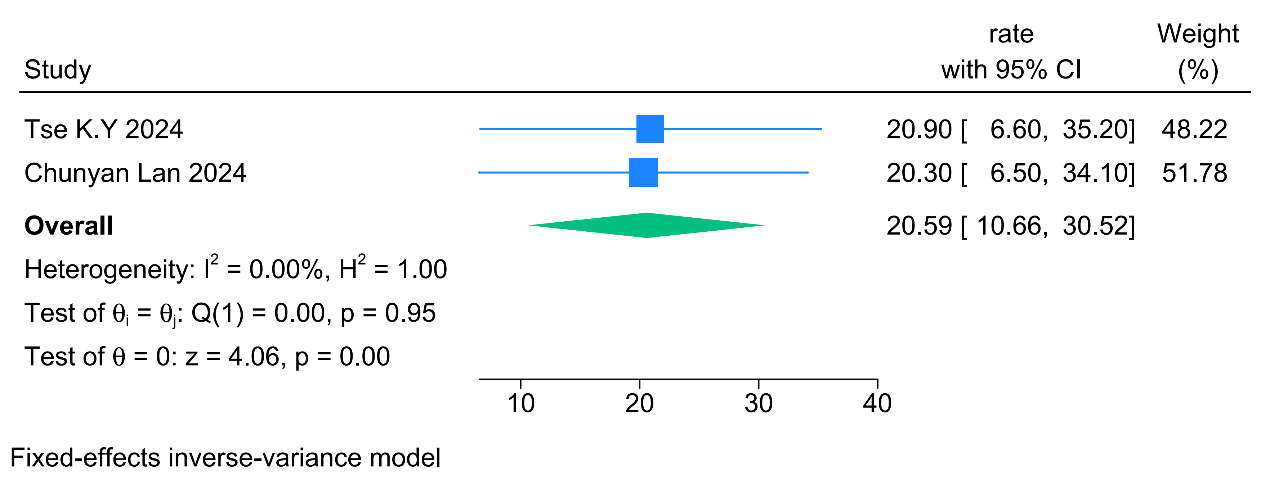


**Supplementary Figure 1.** Forest plot for overall survival (OS) in single‑arm studies.


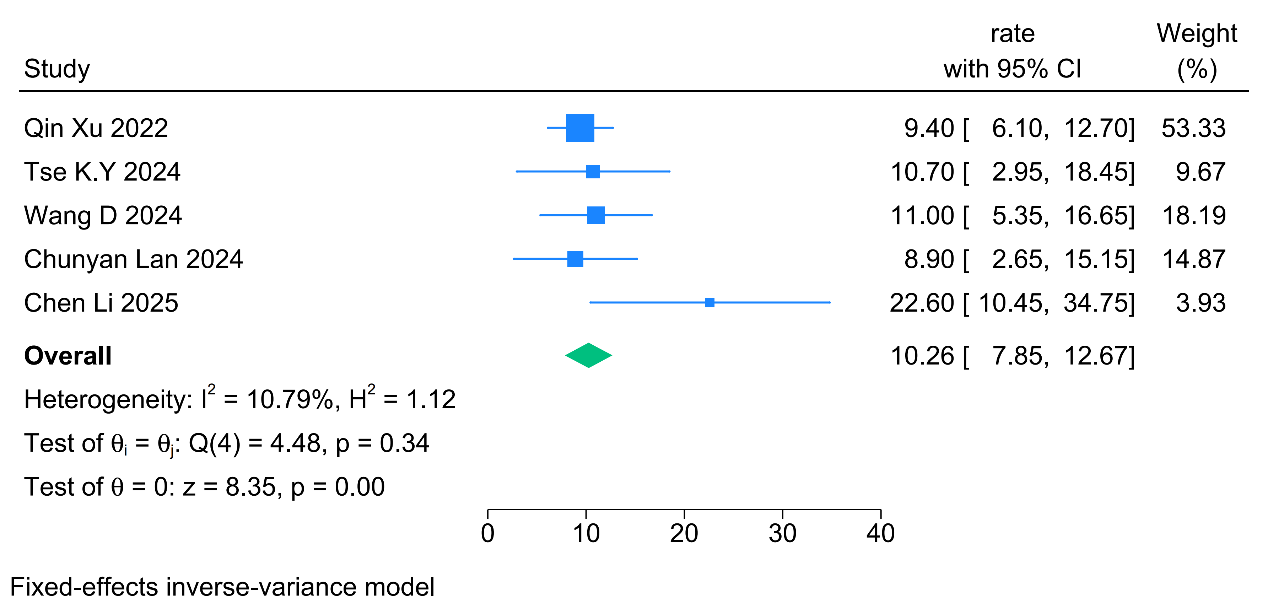


**Supplementary Figure 2.** Forest plot for progression‑free survival (PFS) in single‑arm studies.


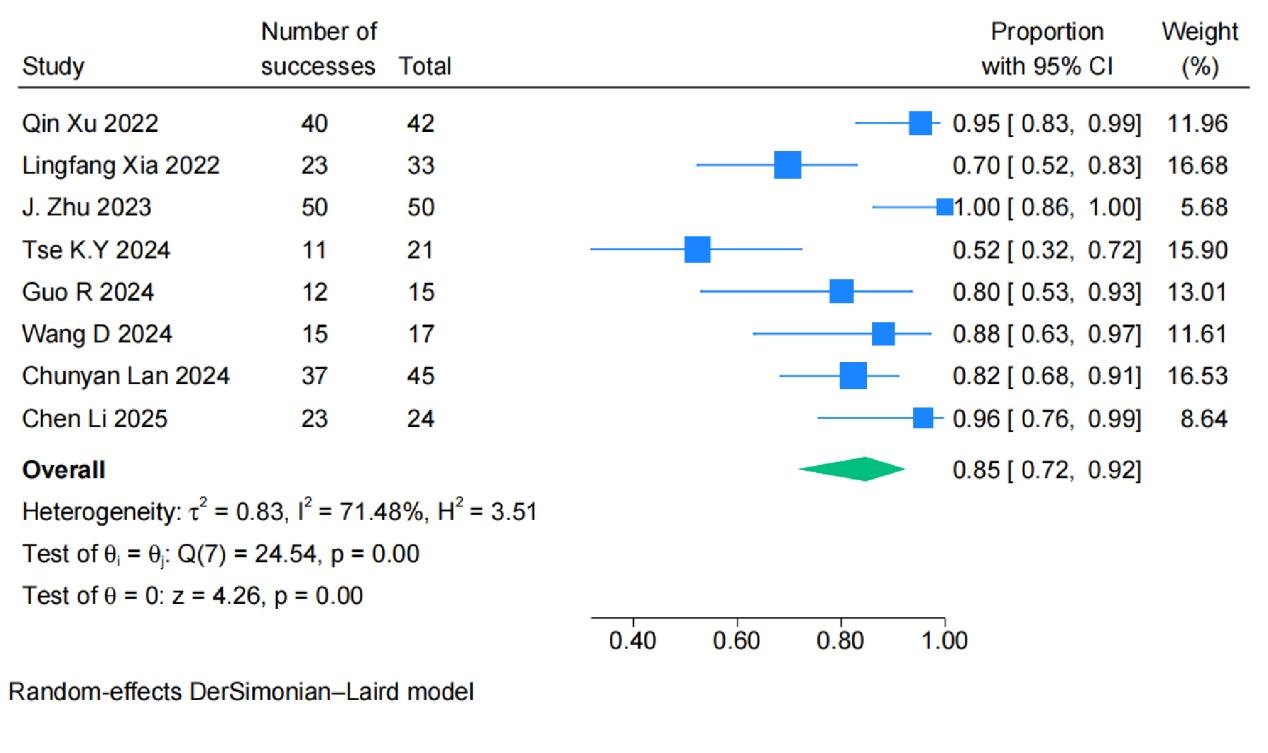


**Supplementary Figure 3.** Forest plot for disease control rate (DCR)in single‑arm studies.

**Supplementary Figure 4.** Funnel plot for the objective response rate (ORR) of the eight single‑arm studies. The pseudo 95% confidence limits are shown as dashed lines. Egger's test P = 0.587.
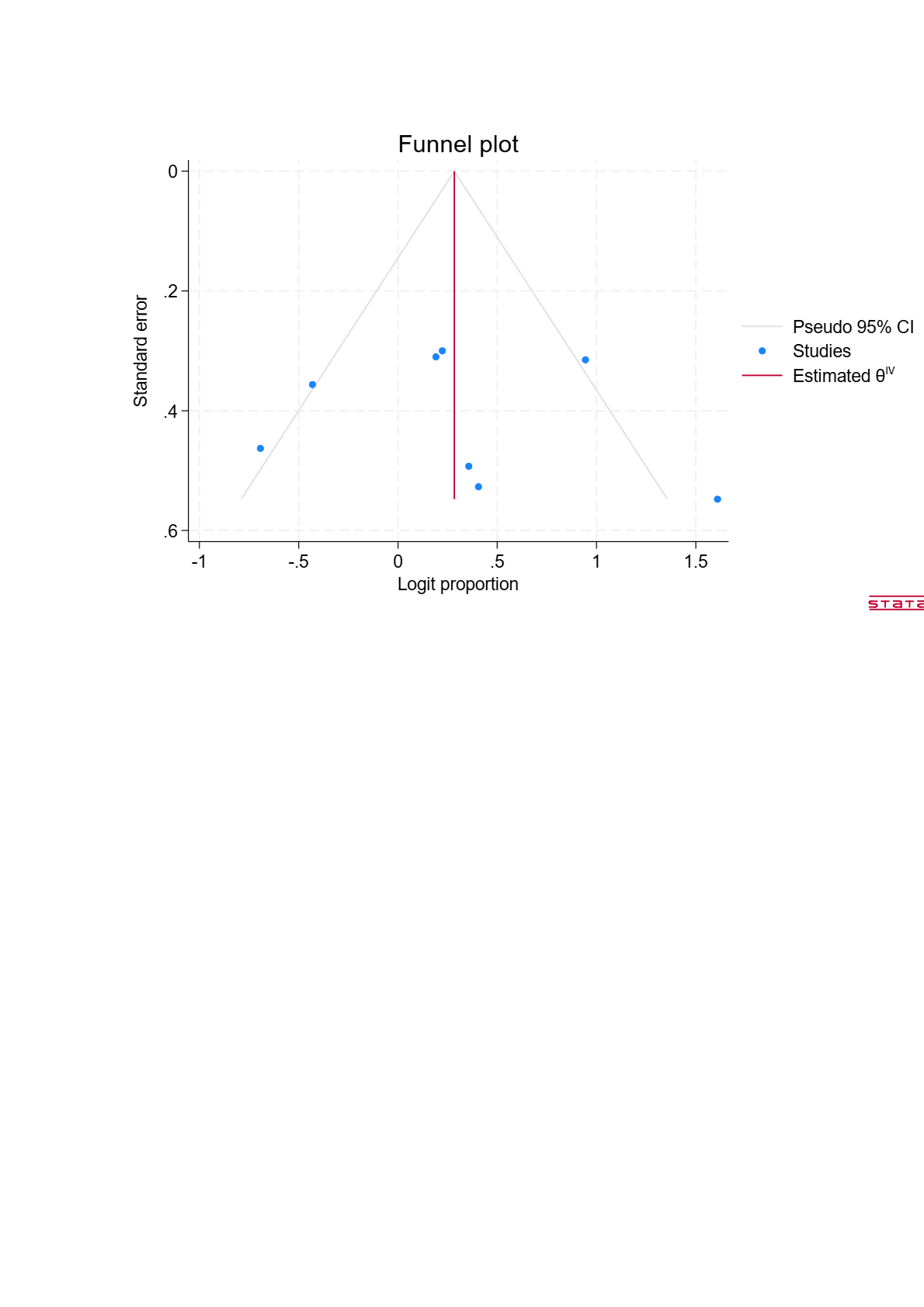

Supplement: Supplementary file 3 [file Supplementaryfile3.docx]
